# Modification of avian pathogenic *Escherichia coli* χ7122 lipopolysaccharide increases accessibility to glycoconjugate antigens

**DOI:** 10.1186/s12934-022-01903-4

**Published:** 2022-09-07

**Authors:** Alexander A. Smith, Ricardo Corona-Torres, Rachel E. Hewitt, Mark P. Stevens, Andrew J. Grant, the Glycoengineering of Veterinary Vaccines Consortium, Brendan Wren, Jon Cuccui

**Affiliations:** 1 Department of Veterinary Medicine, University of Cambridge, Madingley Road, Cambridge, UK; 2 The Roslin Institute and Royal (Dick) School of Veterinary Studies, University of Edinburgh, Easter Bush, Midlothian, UK

**Keywords:** Protein glycan coupling technology, Vaccine, Poultry, Glycoconjugate lipopolysaccharide

## Abstract

**Background:**

Worldwide, an estimated 70.7 billion broilers were produced in 2020. With the reduction in use of prophylactic antibiotics as a result of consumer pressure and regulatory oversight alternative approaches, such as vaccination, are required to control bacterial infections. A potential way to produce a multivalent vaccine is via the generation of a glycoconjugate vaccine which consists of an antigenic protein covalently linked to an immunogenic carbohydrate. Protein-glycan coupling technology (PGCT) is an approach to generate glycoconjugates using enzymes that can couple proteins and glycan when produced in bacterial cells. Previous studies have used PGCT to generate a live-attenuated avian pathogenic *Escherichia coli* (APEC) strain capable of *N*-glycosylation of target proteins using a chromosomally integrated *Campylobacter jejuni pgl* locus. However, this proved ineffective against *C. jejuni* challenge.

**Results:**

In this study we demonstrate the lack of surface exposure of glycosylated protein in APEC strain χ7122 carrying the *pgl* locus*.* Furthermore, we hypothesise that this may be due to the complex cell-surface architecture of *E. coli.* To this end, we removed the lipopolysaccharide O-antigen of APEC χ7122 *pgl*^+^ via deletion of the *wecA* gene and demonstrate increased surface exposure of glycosylated antigens (NetB and FlpA) in this strain. We hypothesise that increasing the surface expression of the glycosylated protein would increase the chance of host immune cells being exposed to the glycoconjugate, and therefore the generation of an efficacious immune response would be more likely.

**Conclusions:**

Our results demonstrate an increase in cell surface exposure and therefore accessibility of glycosylated antigens upon removal of lipopolysaccharide antigen from the APEC cell surface.

## Introduction

The scale of global poultry production is vast, with an estimated 70.7Bn broilers and 1.6Tn eggs produced in 2020 according to the Food & Agriculture Organisation [1]. Infectious diseases pose a significant risk to poultry welfare and productivity. Antibiotics have been used in poultry production, both at sub-therapeutic levels as growth promoters in some countries and for prevention or treatment of bacterial diseases [2]. However, the effectiveness of antibiotics is waning with the evolution of transmissible antibiotic resistance [3, 4]. The most common bacterial pathogens that infect or colonise poultry include avian pathogenic *Escherichia coli* (APEC), *Salmonella enterica*, *Clostridium perfringens* and *Campylobacter jejuni* [5–8]*.* These pathogens are known to either cause severe disease in poultry, thereby impacting animal welfare and productivity, or are zoonotic pathogens that enter the food chain and cause disease in humans. An alternative approach to prevent infection is vaccination. The development of efficacious vaccines could not only prevent avian and zoonotic diseases but also help to combat antibiotic resistance [9]. Multivalent vaccine development is especially appealing due to the potential for immunization against multiple organisms in a single dose, thus reducing labour and cost.

Glycoconjugate vaccines are produced by covalently linking a bacterial polysaccharide (usually capsule or O-antigen) to an immunogenic carrier protein. Glycoconjugate vaccines against bacteria are one of the success stories of modern medicine and have led to a significant reduction in the global occurrence of bacterial meningitis and pneumonia in humans but have not been used for animals. Protein Glycan Coupling Technology (PGCT) is a method that exploits the *C. jejuni N*-linked glycosylation system, encoded by genes within the *C. jejuni pgl* locus*,* to produce glycoconjugates in vivo and promises a low-cost alternative to traditionally made chemically conjugated vaccines [10]. The *pgl* locus contains the genes required to synthesise the *C. jejuni* heptasaccharide glycan and transfer the glycan to an acceptor protein [11, 12]. The oligosaccharide transferase PglB is the enzyme responsible for transferring the glycan from the lipid anchor on which it is synthesised, to an acceptor protein. PglB transfers the glycan to proteins containing the consensus sequence D/E-X_1_-N-X_2_-S/T (where X is not proline) [13]. The integration of the *pgl* locus into the APEC χ7122 genome resulted in exogenous proteins incorporating the D/E-X_1_-N-X_2_-S/T motif being successfully glycosylated, demonstrating the potential of a multivalent live vaccine to be developed using this technology [14]. However, although vaccination of chickens with this strain was able to reduce APEC colonization of the lungs upon experimental challenge, no reduction in *C. jejuni* colonization of the caeca was detected after challenge, indicating that further optimisation of the vaccine strain was required. Importantly, surface display of the antigen was limited in the study, particularly at the body temperature of chickens [14, 15].

An important feature of the *E. coli* cell surface architecture is the lipopolysaccharide (LPS) [16]. We hypothesised that truncation of the LPS by removal of the O-antigen would not only impair virulence and colonisation, but also potentially increase immune cell exposure to the glycosylated heterologous protein [17]. Removal of the immunodominant O-antigen in *Salmonella* by mutation of *rfaH* has been reported to enhance responses to underlying antigens and increase cross-serovar protection [18]. The *wecA* gene (previously termed *rfe*) encodes the O-antigen transferase (undecaprenyl-phosphate α-N-acetylglucosaminyl transferase), which initializes the synthesis of O-antigen polysaccharide and the enterobacterial common antigen (ECA) [19, 20]. Removal of *wecA* results in loss of O-antigen and ECA polysaccharide production [20, 21]. 

In this study we aimed to increase the surface presentation of the glycosylated antigenic protein previously described by Mauri et al. [14]. The APEC χ7122 *pgl* integrant vaccine strain and expression plasmids optimised for in vivo longevity, expression, and glycosylation were used. To assess whether glycosylation efficiency and/or surface presentation was target protein dependent, two different antigenic proteins were selected. The *C. perfringens* NetB toxin and the *C. jejuni* FlpA protein were used as carrier protein glycosylation targets in this study. Previous studies have demonstrated the immunogenicity of NetB and FlpA, making them ideal candidates for use in a multivalent poultry vaccine [22, 23]. The effect of temperature on glycosylation was also assessed, comparing permeabilised glycosylation levels for bacteria grown at 28 °C to those grown at 37 °C or 42 °C.

## Materials and methods

### Bacterial strains, media, and growth conditions

APEC strain χ7122 (O78:H9) and *E. coli* DH5α (New England Biolabs) (Table 1) were cultured on Luria Bertani (LB) agar plates for 16 h at 37 °C. Alternatively, strains were grown for 16 h in LB broth at 37 °C; cultures were shaken at 200 rpm in LB medium at 37 °C. Where necessary, media were supplemented with the appropriate antibiotic for selection (ampicillin 100 μg/ml, kanamycin 50 μg/ml, chloramphenicol 25 μg/ml, and gentamicin 20 μg/ml).

**Table 1:** Bacterial strains and plasmids used in this study

Strain or plasmid	Relevant genotype or description	Source or references
Strain		
APEC χ7122	Wild type strain	[29]
APEC χ7122 *pgl*^+^	*pgl* integrant, kan^r^	[14]
APEC χ7122 *pgl*^+^ Δ*wecA::cat*	*pgl* integrant with *wecA* deleted and replaced with *cat*, kan^r^, cm^r^	This study
Plasmid		
pFPV25.1-G-NetB(10)	Plasmid stably maintained in APEC that expresses NetB with 10 PglB target sites under the control of a constitutive *rpsM* promoter, amp^r^	[14]
pFPV25.1-FlpA-10GT	Plasmid stably maintained in APEC that expresses FlpA with 10 PglB target sites under the control of a constitutive *rpsM* promoter, amp^r^	This study
pBADλred	λRed recombineering plasmid, amp^r^	[26]

### Recombinant DNA techniques

Standard methods were used for molecular cloning. Chromosomal and plasmid DNA purifications were performed using commercial kits following the manufacturers’ instructions (New England Biolabs). DNA concentration and purity were measured using a Nanodrop ND-1000 spectrophotometer.

### Construction of *wecA* gene deletion mutant

The APEC *wecA* gene deletion mutant was constructed by allelic replacement with a chloramphenicol acetyl transferase (*cat*) resistance cassette using a modification of the ET cloning procedure [24, 25] as previously described [26]. The addition of a chloramphenicol resistance cassette allowed for the selection of successful recombination events. A fragment containing the DNA to be integrated onto the chromosome was amplified from DNA containing the *cat* gene, using primers wecA:Cm_del_F (GGTCTTCGTGGTTATACTTCTGCTAATAATTTTCTCTGAGAGCGCATTACACGTCTTGAGCGATTG) and wecA:Cm_del_R (TTCGGCCGGTTTCCCAGGCATTGGTTGTGTCATCACATCCTTAGCCATGGTCCATATGAA). Nucleotides underlined are within the *cat* cassette, whereas nucleotides that are not underlined are present in the APEC χ7122 genome. Primers were designed with a 40 bp overhang homologous to the flanking region of *wecA* to allow for homologous recombination. The PCR product was further amplified using wecA_F (GGTCTTCGTGGTTATAC) and wecA_R (TTCGGCCGGTTTCCCAGGC) to generate enough DNA for recombination. Approximately 1 µg of linear PCR product was used for integration onto the chromosome using a modification of the λRed method as previously detailed [26, 27]. Expression of λRed recombinase from plasmid pBADλred [26] was induced with 0.2% l-arabinose. Electroporation was used to introduce the PCR amplicon into target cells, followed by a 3-h incubation in SOC medium. Transformants were then plated onto selective media. Loss of the pBADλred helper plasmid was performed using repeat passage and MAST ID Intralactam strips (MAST Diagnostics) to screen for the absence of beta-lactamase in bacterial colonies. The resultant gene deletion mutants were confirmed by PCR. Additionally, these PCR products were verified by Sanger sequencing (using primers wecA_1 and wecA_2). The gene deletion mutant was confirmed via whole genome sequencing (microbesNG). BAM files were generated using bowtie2 and aligned to the APEC χ7122 genome using Artemis to confirm deletion of *wecA* and replacement with *cat (NCBI accession JANRGZ01)* [28–30].

### Generation of pFPV25.1 plasmids

The pFPV25.1_flpA_10GT construct is based on plasmid pFVP25.1 which is stably maintained during APEC infection of chickens [31] and includes a ribosome binding site, a PelB signal sequence, the *C. jejuni flpA* sequence codon optimised for *E. coli*, five repeats of the *N-*glycosylation site DQNAT at each side of *flpA* and a *C-*terminal 6xHis tag. The fusion was commercially synthesised (GeneArt, ThermoFisher, UK) and subcloned into pFPV25.1 (Valdivia, 1996) using restriction sites XbaI and HindIII. pFPV25.a-G-NetB(10) has similar composition but with the *C. perfringens netB* coding sequence and has been described previously [14]. APEC χ7122, APEC χ7122 *pgl*^+^ and APEC χ7122 *pgl*^+^ Δ*wecA* were transformed with pFPV25.1_flpA_10GT and pFPV25.1-G-NetB(10) using electroporation. Cells were made electrocompetent via repeat washing with ice cold 10% glycerol.

### Immunofluorescent staining

Immunofluorescent staining was used to detect surface expression of glycosylated heterologous antigenic proteins, both qualitatively and quantitatively, using flow cytometry and confocal microscopy. Preparation of bacterial cultures for staining was completed following a previously described protocol with some modifications [32]. Briefly, bacterial cultures were grown overnight in LB with appropriate antibiotics and fixed with 4% paraformaldehyde. Samples requiring permeabilization were treated with 70% ethanol, lysozyme (25 µg/ml) and DNAase (50 U/ml). Permeabilization allows for the assessment of total protein production, as not all recombinant protein was transported to the cell surface. Between each step, the samples were washed three times with PBS. Following fixation and enzyme treatment (if required), samples were blocked with 0.1% BSA and stained with the lectin soybean agglutinin (SBA) from Glycine max conjugated to Alexa Fluor™ 647, 6x-His Tag Monoclonal Antibody (HIS.H8) Alexa Fluor 488 and Hoechst nuclear stain.

### Confocal microscopy

Samples prepared for confocal microscopy were fixed on silane-coated slides to aid bacterial cell adhesion. Data was acquired on an Axio Examiner Z1 microscope equipped with a Zeiss LSM780 scanhead, using an 63 × oil immersion lens. 405 nm, 488 nm and 633 nm lasers were used to excite the Hoechst nuclear stain, Alexa Fluor 488 and Alexa Fluor 647 conjugates, respectively. Data were collected and the images analysed using Zeiss Zen software.

### Flow cytometry

To reduce clumping prior to analysis, bacterial cells were passed through a 35 µm-mesh strain filter. Data for 500,000 events was collected when there were enough bacterial cells present. Single stained compensation tubes for each fluorophore and an unstained sample were also acquired in order to set laser volatges and compensate for any spectral overlap. Four biological repeats were performed, unless specified otherwise. One data point was collected for APEC χ7122 (negative control). Data were acquired on a CyAn™ ADP Cytometer equipped with 405 nm, 488 nm and 635 nm lasers in standard configuration, and analysed on FlowJo software.

### Statistical analyses

Two-tailed, unpaired Student’s T-tests were performed to calculate statistical significance. For flow cytometry data, all samples within a group (permeablised or non-permeablised) were compared. Corresponding samples between groups at the same temperature (for example, APEC χ7122 *pgl*^+^  + pFPV25.1-flpA-10GT non-permeabilised *vs* APEC χ7122 *pgl*^+^  + pFPV25.1-flpA-10GT permeabilised) were also compared. When comparing data sets with one data point against those with multiple data points, a one-sample T-test was performed. A p-value ≤ 0.05 was considered to be statistically significant.

## Results and discussion

Our previous studies investigating the use of glycoengineered vaccines to prevent both APEC and *C. jejuni* colonisation of chickens have had limited success [14, 33, 34]. It has previously been demonstrated that APEC χ7122 *pgl*^+^ is capable of glycosylating heterologous proteins when the D/E-X_1_-N-X_2_-S/T glycosylation motif has been incorporated into the amino acid sequence of the protein [14]. However, host (chicken) recognition of the *C. jejuni* heptasccharide glycan has been shown to be variable [14, 35]. In vivo chicken studies have shown little immunological recognition of glycosylated antigenic proteins delivered via live bacterial vaccines in poultry [14]. This study aimed to elucidate mechanisms which may be inhibiting the development of a robust immune response in these animals and therefore limiting the efficacy of glycoconjugate vaccines.

**Fig. 1:**
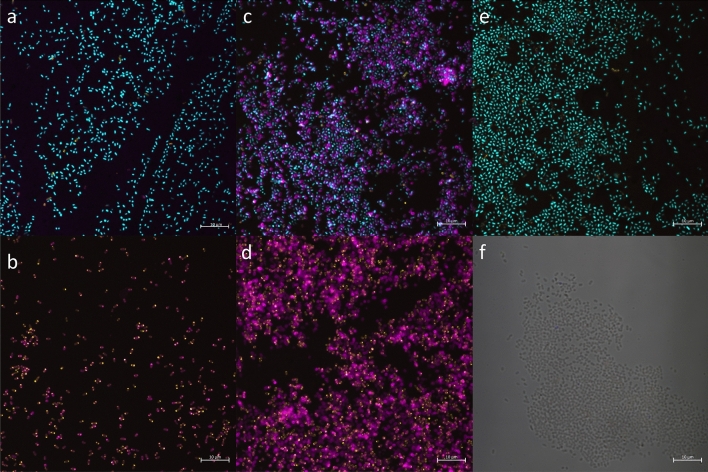
Confocal microscopy images of APEC χ7122 *pgl*^+^ expressing glycosylatable FlpA. Bacteria were stained with lectin SBA from Glycine max (soybean) Alexa Fluor™ 647 conjugate (magenta), 6xHis-tag monoclonal antibody (HIS.H8) Alexa Fluor 488 (yellow) and Hoechst nuclear stain (cyan). **A** APEC χ7122 *pgl*^+^  + pFPV25.1-flpA-10GT, **B** APEC χ7122 *pgl*^+^  + pFPV25.1-flpA-10GT with permeabilization, **C** APEC χ7122 *pgl*^+^ Δ*wecA::cat* + pFPV25.1-flpA-10GT, **D** APEC χ7122 *pgl*^+^ Δ*wecA::cat* + pFPV25.1-flpA-10GT with permeabilization, **E** APEC χ7122 *pgl*^+^, **F** APEC χ7122 with permeabilization. Images B, D and F lack Hoechst nuclear stain due to permeabilization requiring DNase treatment. Scale bar = 10 µm

A key step in the development of a glycoconjugate vaccine-specific immune response is host exposure to the glycosylated antigenic protein. To this end, transportation of antigenic protein to the periplasm and surface expression was assessed qualitatively using antibody staining and confocal microscopy (Fig. 1). An APEC χ7122 strain containing the *C. jejuni pgl* locus, termed APEC χ7122 *pgl*^+^*,* was transformed with pFPV25.1_flpA_10GT, a plasmid containing the gene sequence of *flpA*, encoding the *C. jejuni* fibronectin-binding adhesin FlpA. pFPV25.1 has been shown to be stable during infection with APEC O1 and O2 [31]. Five sequential glycosylation motifs were incorporated before and after the FlpA amino acid sequence. The presence of the glycosylation motif ensures the protein is a target for *N*-linked glycosylation via the chromosomally integrated *pgl* locus. A PelB leader sequence is incorporated into the protein sequence to enable transportation of the protein to the periplasm where glycosylation occurs. Lectins are glycoproteins that strongly bind to specific glycans, and fluor-tagged lectins can be used to identify proteins that have successfully been glycosylated. A 6xHis-tag was also incorporated into the *flpA* sequence to enable protein quantity and location to be assessed. Antibodies specific for FlpA were not available, therefore the 6xHis tag present on each protein was used as a proxy for staining.

APEC χ7122 *pgl*^+^  + pFPV25.1_flpA_10GT (Fig. 1A) showed low amounts of surface presentation of the antigen, demonstrated by minimal staining for both the glycan and 6xHis-tag. Upon permeabilization, stained protein was observed, demonstrating the successful expression and glycosylation of FlpA within the bacterium (Fig. 1B). This data suggests that either the target protein is not being transported to the cell surface, and/or antibody accessibility of the target protein is hindered. To address these hypotheses, the O-antigen of APEC χ7122 *pgl*^+^ was removed. The *wecA* gene*,* encoding the O-antigen transferase, was deleted from APEC χ7122 *pgl*^+^ via homologous recombination. The resulting strain, APEC χ7122 *pgl*^+^ *ΔwecA::cat,* demonstrated increased levels of staining with fluorescently-labelled SBA (Fig. 1C) compared to APEC χ7122 *pgl*^+^ (Fig. 1A), suggesting the presence of complete O-antigen has an inhibitory effect on antibody binding to the glycoconjugate. Conversely, anti-histidine staining was not increased. The lack of 6xHis-tag staining may be due to the 6xHis-tag being embedded in the cell membrane. The increase in SBA lectin staining in the absence of O-antigen supports the hypothesis that host immune cell access to the antigenic protein is hindered due to the surface LPS. In both APEC χ7122 *pgl*^+^ and APEC χ7122 *pgl*^+^ *ΔwecA::cat* containing pFPV25.1_flpA_10GT, the higher quantity of glycosylated protein upon permeabilization of the cell wall remains consistent (Fig. 1B and D). Low levels of background staining were observed in APEC χ7122 *pgl*^+^ (Fig. 1E), which could suggest glycosylation of native proteins at low levels. This background fluorescence might be explained by the presence of the D/E-X_1_-N-X_2_-S/T glycan acceptor motif naturally occurring throughout the APEC χ7122 genome. Indeed, this motif can be found within 268 proteins of APEC χ7122, and it is possible that these proteins are targets of glycosylation. Further analysis would be required to validate this hypothesis.

**Fig. 2:**
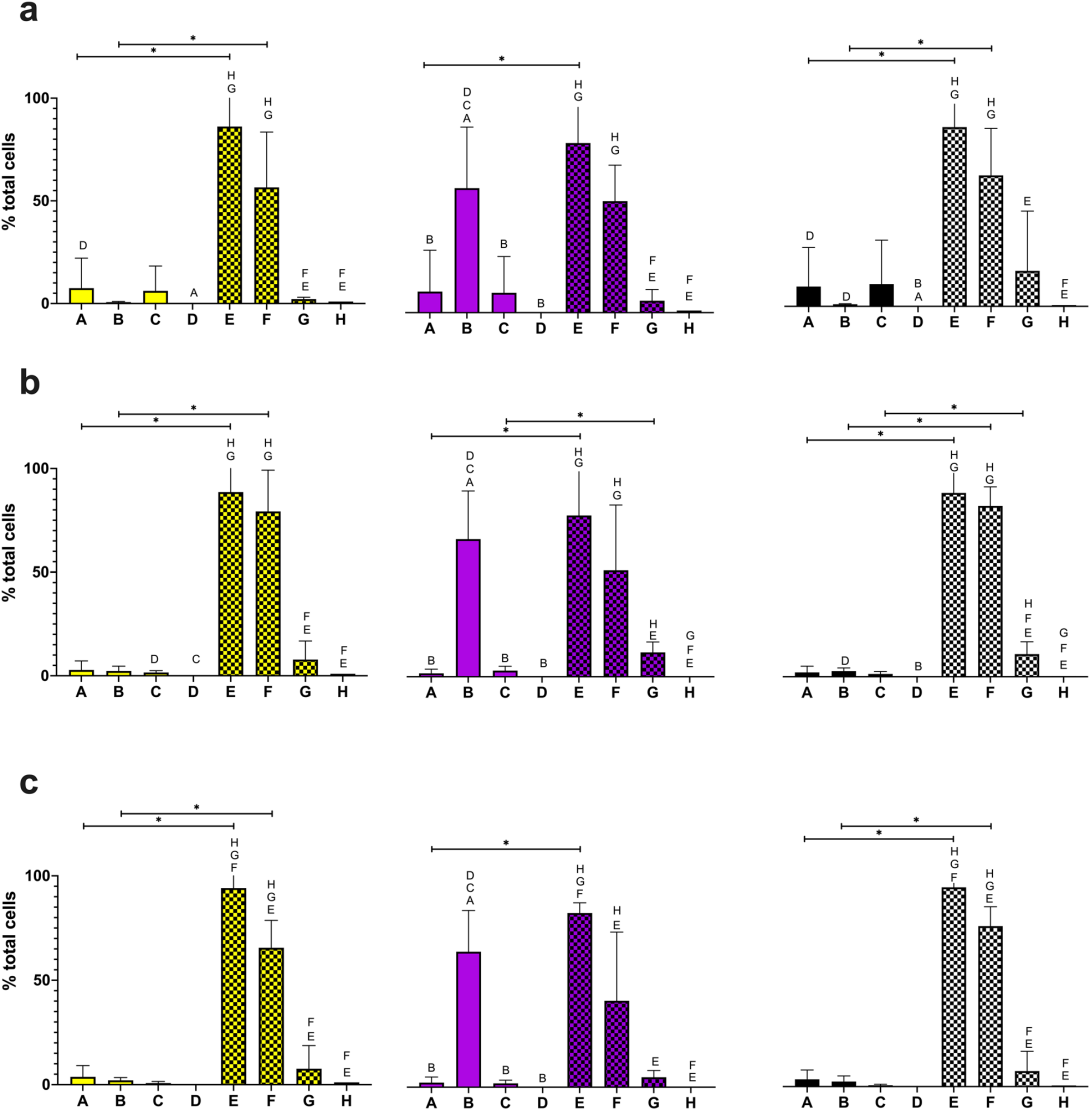
Graphs showing FlpA glycoconjugate staining at various temperatures and permeability states. Percentage of total bacterial cells positive for anti-6xHis-tag staining (yellow), lectin staining (purple) and anti-6xHis-tag + lectin staining (black), empty bars = non-permeabilized, hatched bars = permeabilized, at (a) 28 °C, (b) 37 °C and (c) 42 °C. **A** APEC χ7122 *pgl*^+^  + pFPV25.1-flpA-10GT, **B** APEC χ7122 *pgl*^+^ Δ*wecA::cat* + pFPV25.1-flpA-10GT, **(C)** APEC χ7122 *pgl*^+^, **D **APEC χ7122, **E** APEC χ7122 *pgl*^+^  + pFPV25.1-flpA-10GT with permeabilization, **F** APEC χ7122 *pgl*^+^ Δ*wecA::cat* + pFPV25.1-flpA-10GT with permeabilization, **G** APEC χ7122 *pgl*^+^ with permeabilization, **H** APEC χ7122 with permeabilization. Statistically significant differences between samples (within unpermeabilized and permeabilized groups) are annotated with letters indicating the group(s) for which significance is observed, statistically significant differences between groups are annotated with lines. Two-tailed, unpaired T-test. P < 0.05. Expression for each sample within each group is compared. Expression between groups for only the equivalent sample is compared. N = 4 biological repeats

**Fig. 3:**
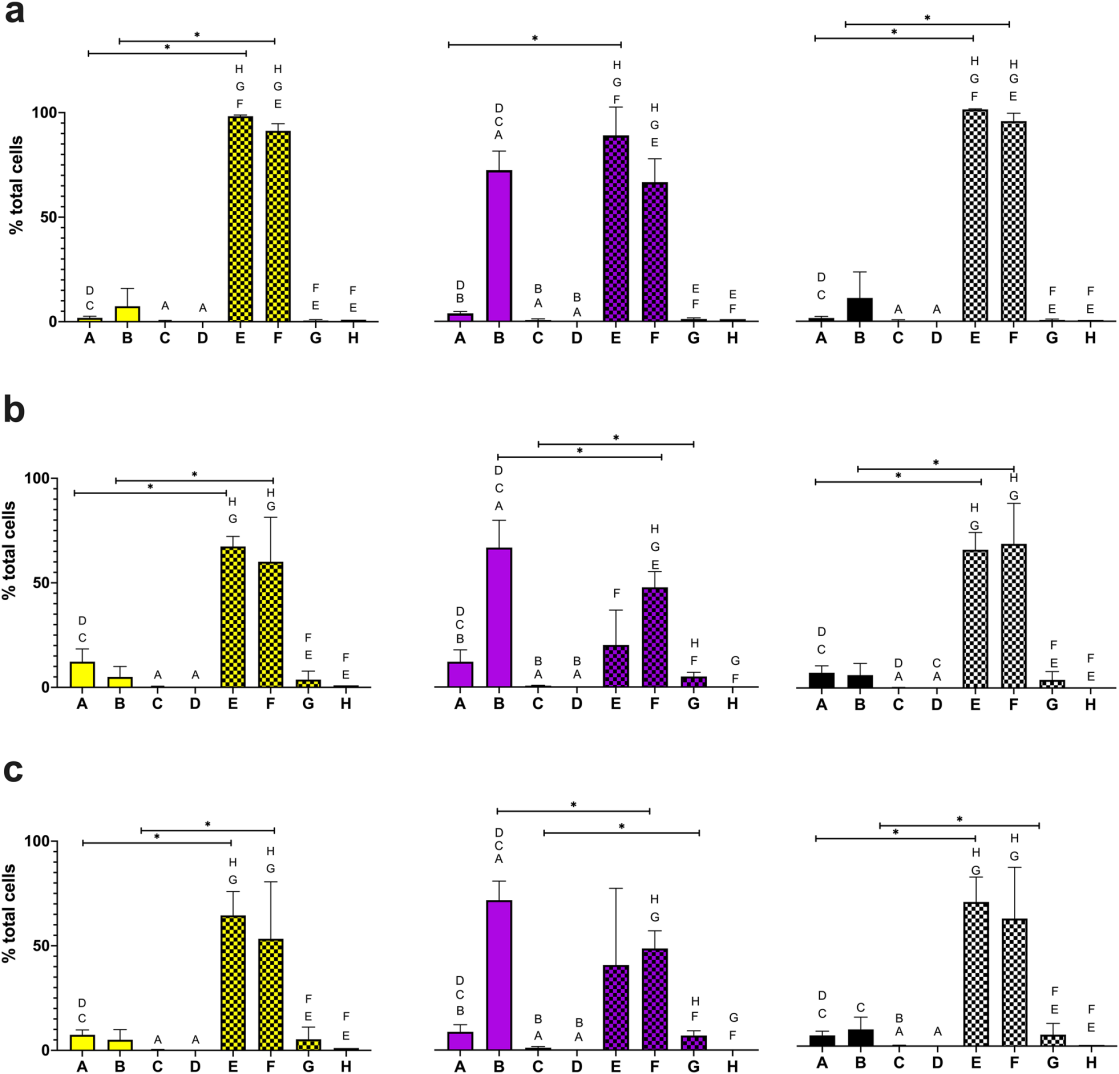
Graphs showing NetB glycoconjugate staining at various temperatures and permeability states. Percentage of total bacterial cells positive for anti-6xHis-tag staining (yellow), lectin staining (purple) and anti-6xHis-tag + lectin staining (black), empty bars = non-permeabilized, hatched bars = permeabilized, at (a) 28 °C, (b) 37 °C and (c) 42 °C. **A** APEC χ7122 *pgl*^+^  + pFPV25.1-G-NetB(10), **B** APEC χ7122 *pgl*^+^ Δ*wecA::cat* + pFPV25.1-G-NetB(10), **C** APEC χ7122 *pgl*^+^, **D** APEC χ7122, **E** APEC χ7122 *pgl*^+^  + pFPV25.1-G-NetB(10) with permeabilization, **F** APEC χ7122 *pgl*^+^ Δ*wecA::cat* + pFPV25.1-G-NetB(10) with permeabilization, **G** APEC χ7122 *pgl*^+^ with permeabilization, **H** APEC χ7122 with permeabilization. Statistically significant differences between samples (within unpermeabilized and permeabilized groups) are annotated with letters indicating the group(s) for which significance is observed, statistically significant differences between groups are annotated with lines. Two tailed, unpaired T-test. P < 0.05. Expression for each sample within each group is compared. Expression between groups for only the equivalent sample is compared. N = 4 biological repeats

Following qualitative analysis via confocal microscopy, quantitative analysis was performed using flow cytometry (Figs. 2 and 3). Here, two target antigens were compared: FlpA and NetB. The use of alternative antigens from different organisms (*i.e*., FlpA from *C. jejuni*, and NetB from *C. perfringens*) allows for the development of a multivalent vaccine. The effect of temperature was also assessed, with each culture being grown at 28ºC, 37ºC and 42ºC. The temperatures represent the optimal glycosylation temperature, optimal *E. coli* growth temperature and avian body temperature, respectively [15]. Bacterial cells were examined for the presence of the 6xHis-tag, glycan and both 6xHis-tag and glycan.

Analysis of single cells using flow cytometry mirrored the qualitative results observed with confocal microscopy. In all non-permeabilized conditions, the proportion of glycan-positive cells was greater in the APEC χ7122 *pgl*^+^ Δ*wecA::cat* + plasmid (pFPV25.1-flpA-10GT or pFPV25.1-G-NetB(10)) *vs* the APEC χ7122 *pgl*^+^  + plasmid (pFPV25.1-flpA-10GT or pFPV25.1-G-NetB(10)) further supporting the hypothesis that the O-antigen hinders antigen accessibility. Interestingly, higher numbers of glycan-positive cells were seen in the APEC χ7122 *pgl*^+^ compared to the wild type APEC χ7122, further suggesting that native APEC χ7122 proteins could be undergoing glycosylation (*i.e*., the protein products of genes on the APEC genome that by chance contain the glycan acceptor domain). The absence of significant reactivity of the antibody against 6xHis until the cells are permeabilised may be a consequence of the C-terminal location of the tag and topology of the proteins in the membrane.

As demonstrated in previous studies, we observed that glycosylation of NetB was temperature dependent (P < 0.05) [15]. However, a temperature-dependent effect was not observed with FlpA glycosylation. The efficiency of NetB glycosylation was higher at 28ºC compared to both 37ºC and 42ºC. The effect of temperature-dependent glycosylation may have an impact on glycoconjugate vaccine design. The carbohydrate portion of a glycoconjugate vaccine has a major role in the development of an efficacious immune response [36]. If glycosylation efficiency is lower at higher temperatures, resulting in the presentation of protein without carbohydrate, then ensuring enough target antigens are glycosylated at the temperatures experienced in vivo is crucial. An alternative approach to ensure that optimal glycosylation is achieved is to grow the bacteria in vitro, at the optimal glycosylation temperature, and then repeatedly administer the bacteria. This may be necessary to generate a robust, prolonged immune response.

An alternative explanation to the phenomenon observed in this study, is that heptasaccharide glycan is binding to the truncated LPS core as well as D/E-X_1_-N-X_2_-S/T tagged proteins [37]. This strategy has previously been used to fuse heptasaccharide glycan to the LPS core using an O-antigen ligase dependant pathway. However, this was performed in an *E. coli* K-12 O-antigen polymerase mutant, compared to the O-antigen transferase mutant described in this study [37].

## Conclusion

In this study we have described the generation and in vitro assessment of an APEC χ7122 *pgl*^+^ Δ*wecA::cat* strain with increased expression/presentation of surface expressed glycoconjugates compared to APEC χ7122 *pgl*^+^*.* Initially, confocal microscopy was used to qualitatively assess and confirm disparities in surface presentation of glycosylated proteins. This observation was further validated using a quantitative flow cytometry approach. Permeabilization revealed that APEC χ7122 *pgl*^+^ and APEC χ7122 *pgl*^+^ Δ*wecA::cat* were capable of producing intracellular glycosylated FlpA and NetB, however APEC χ7122 *pgl*^+^ Δ*wecA::cat* demonstrated significantly more surface-exposed glycan. This data suggests that, in previous studies using an in vivo chicken model of infection, APEC χ7122 *pgl*^+^ may not be sufficient in provoking a carbohydrate-stimulated immune response, potentially due to the blocking of surface expressed glycoconjugate due to the O-antigen [14]. Therefore, strains lacking O-antigen and/or other cell surface architecture may be more suited to glycoconjugate vaccine development and warrant further in vitro and in vivo investigation for this purpose.

## Data Availability

The data that support the findings of this study are available from the corresponding author [AJG], upon reasonable request.
